# Effects of perceptual variables on numerosity comparison in 5–6-year-olds and adults

**DOI:** 10.3389/fpsyg.2013.00431

**Published:** 2013-07-24

**Authors:** Midori Tokita, Akira Ishiguchi

**Affiliations:** Graduate School of Humanities and Sciences, Ochanomizu UniversityTokyo, Japan

**Keywords:** approximate number representation, weber fraction, method of constant stimuli development

## Abstract

Although a critical issue in the debate over the existence of abstract numerical representation, it remains unclear whether and how perceptual variables affect numerosity judgment and how they change across development stages. In this research, we examine the effects of perceptual variables on approximate numerosity comparison in 5–6-year-olds and adults using the identical experimental procedure. In the assessment of the effect of the perceptual variables, we measured precision (i.e., Weber fraction) and accuracy (i.e., point of subjective equality; PSE) of the numerosity comparison. In Study 1, we tested how the manipulation of the cumulative element area would affect approximate numerosity comparison. The results showed that Weber fractions increased and the size of bias enlarged in the large element condition in both adults and 5–6-year-olds. In study 2, we tested how the manipulation of the array area would affect the precision and accuracy of approximate numerosity comparison. The results demonstrated that Weber fractions increased and the size of bias enlarged in the large array condition in both adults and 5–6-year-olds. Overall, our results suggest that the effect of perceptual variables on 5–6-year-olds is qualitatively similar to that on adults. In addition, we also tested whether the performance of approximate comparison correlated with the initial numerical skill in 5–6-year-olds to reveal least relationship between them.

## Introduction

Many studies in the behavioral, neurophysiological, and brain imaging fields have suggested a dedicated mechanism for approximate numerical representation shared across development stages and across species (e.g., Gallistel and Gelman, 1992; Dehaene, 1997; Eger et al., 2003; Feigenson et al., 2004; Nieder and Miller, 2004; Cappelletti et al., 2007; Cantlon et al., 2009; Nieder and Dehaene, 2009; Piazza, 2010). Consistent with this argument, it has been noted that approximate numerical representation may be crucial to the acquisition of symbolic number representations that are uniquely human (Halberda and Feigenson, 2008; Halberda et al., 2008; Mussolin et al., 2012). Several studies show that the development of symbolic numerical knowledge relies on the representation of approximate numerosities, and that the ability of approximate numerosity increases with age and throughout the school years (Halberda et al., 2008; Piazza et al., 2013). However, certain researchers have also challenged the idea of the abstract numerical representation by presenting various empirical data that demonstrate methodological and theoretical limitations (e.g., Kadosh et al., 2008; Kadosh and Walsh, 2009; Tokita and Ishiguchi, 2012). For example, Kadosh and Walsh (2009) prompted extensive investigation of number representation processes in the behavioral and neurophysiological fields, suggesting the existence of a general magnitude system serving all sorts of quantity. The general magnitude system predicts that the non-numerical magnitude such as cumulative area, luminance, or presentation duration may interact with the performance of numerical judgments (e.g., Walsh, 2003; Lourenco and Longo, 2010).

One of the critical issues in the debate over the existence of abstract representation is whether perceptual variables affect numerosity judgment. Proponents of the dedicated system for abstract numerical representation have claimed that the approximate numerical system would be capable of representing the numerosity of any set of discrete elements, whether they were events or items, presented sequentially or simultaneously, independent of both the stimuli's spatial arrangements and the individual elements' attributes such as size, color, and shape. In contrast, skeptics of the system have claimed that perceptual variables could affect numerosity comparison, especially in infants and young children. Certain research predicts that the non-numerical information such as surface area, contour length, or spatial arrangement of the elements in the set could be used in estimation or judgments of numerosity (e.g., Clearfield and Mix, 2001; Mix et al., 2002; Rousselle et al., 2004; Rousselle and Noel, 2008).

Despite extensive research, however, it remains unclear whether and how perceptual variables affect numerosity judgment and how they change across development stages. Several studies found that numerical processing in infants and young children is affected by perceptual variables such as element area, contour length, and density, suggesting that their judgment is not solely based on the number of objects. It has been demonstrated that infants and young children preferentially attend to perceptual variables of sets rather than the number of objects in a set (e.g., Clearfield and Mix, 2001; Mix et al., 2002; Rousselle et al., 2004). For example, Rousselle et al. (2004) demonstrated that the performance of numerical tasks in 3-year-olds deteriorated when the number of elements and the continuous variables were controlled. In contrast, other studies have claimed that infants are not affected by the area size or contour length of the element and can judge solely on the basis of the number of elements (e.g., Xu et al., 2005; Cordes and Brannon, 2008). The authors of those studies have asserted that the number may be more relevant than perceptual variables such as area and contour length and may be spontaneously represented.

In adult studies, however, many empirical investigations have found that perceptual variables such as the size of elements, array area, and spatial arrangement of elements affect the precision and/or accuracy of the numerosity judgment (e.g., Allik and Tuulmets, 1991; Hurewitz et al., 2006; Tokita and Ishiguchi, 2010; Gebuis and Reynvoet, 2012). For example, Tokita and Ishiguchi (2010) tested the effects of element size and array area in numerosity comparison by using a wide range of numbers of elements. They showed that element size and array area affect precision and accuracy in numerosity discrimination tasks. Other research has shown that the spatial arrangements of dots could affect the numerosity judgment (e.g., Ginsburg, 1991; Sophian and Chu, 2008; He et al., 2009). For example, Sophian and Chu (2008) demonstrated that numerosity judgments were affected by the amount of open space in the arrays being compared.

What really is the effect of perceptual variables in young children? Does the effect differ from that in adults? How does the effect change across development stages? In the present research, we examine whether and how perceptual variables affect approximate numerosity comparison in 5–6-year-olds and adults. The present research has two unique investigative features that differ from previous research. First, for the perceptual variables' effect, we measured both precision (i.e., variability of the observer's response or estimation) and accuracy (i.e., whether the number of elements was overestimated or underestimated as compared with the objective numerical value) using a psychophysical method. Conventionally, the effect of perceptual variables has been measured by either accuracy or precision, and few studies have tested both. Because perceptual variables could affect both the precision and accuracy of task performance, we considered it necessary to measure both in a particular experiment. We used the method of constant stimuli in which observers decided in each trial which visual array—a standard or a comparison array—had more elements. To test the precision, we derived Weber fractions that indicate the observer's variance of numerosity judgment. Both behavioral and neurobiological evidence reported that numerosity comparison obeys the Weber law: discriminability depends on the ratio of the numerosity to be compared (e.g., Burgess and Barlow, 1983; Huntley-Fenner, 2001; Piazza et al., 2004; Jordan and Brannon, 2006; Halberda and Feigenson, 2008; Cantlon et al., 2009). To test the accuracy of the numerical comparison, we derived the point of subjective equality of numerical values (PSE). The second unique feature of our study is that we used identical experimental procedures and stimuli conditions for 5–6-year-olds and adults. Few studies to date have attempted to test the perceptual variables both in young children and adults using the same procedures.

We introduced two types of perceptual variables: the size of the element area and that of the array area. The element area refers to the cumulative area of the elements in a set. The array area refers to the envelope space where the elements in a set are laid out. In the first study, we examined whether and how the manipulation of the cumulative element area affects the numerosity comparison. In the second study, we examined whether and how the manipulation of the array area affects the numerosity comparison. If the size of the cumulative area or the array area affects the numerosity comparison task, Weber fractions would vary across perceptual conditions and/or the PSE would deviate from the objective numerical value, suggesting dissociation between the objective number of elements and perceived numerosity. Following the comparison task, 5–6-year-olds performed a non symbolic numerical matching task for the assessment of their acquisition of number concept and a symbolic numerical matching task for the assessment of their symbolic numerical knowledge.

In addition, we examined the relationship between approximate comparison and the symbolic numerical knowledge (symbolic numerical knowledge) in 5–6-year-olds in each study. Whether the ability of approximate numerical representation is related to early numerical knowledge such as exact counting and symbolic numerical knowledge is another important issue for numerical research; however, conclusive evidence has not been demonstrated. Certain studies have found that approximate number representation skill relates to symbolic numerical knowledge (Halberda and Feigenson, 2008; Piazza, 2010; Lourenco et al., 2012). Others, however, have shown that the there is no relationship between them, suggesting that non-verbal number knowledge would not be a basis for exact counting and early symbolic numerical ability (Rousselle and Noel, 2008; Soltesz et al., 2010). We tested whether the ability demonstrated in the approximate numerosity representation predicts the initial numerical skill and the approximate comparison performance would correlate with the performance of symbolic numerical matching task.

## Study 1

In the first study, we examined whether and how the manipulation of the cumulative element area affects the numerosity comparison. If the size of the cumulative area affects the numerosity comparison task, Weber fractions would vary across perceptual conditions and/or the PSE would deviate from the objective numerical value, suggesting dissociation between the number of elements and perceived numerosity. Following the comparison task, 5–6-year-olds performed a non symbolic numerical matching task and a symbolic numerical matching task.

### Method

#### Participants

Twenty children (mean age 5.9 years, *SD* = 0.28 years; range 5.5–6.3 years; 9 females, 11 males) and sixteen adults (mean age 26.8 years, *SD* = 4.2 years; range 20–37 years; 12 females, 4 males) participated. Informed consents from a parent of each child were obtained prior to the experiment. None had a statement of special educational needs. All children were recruited though kindergartens and received a sticker to thank them for taking part. All adult participants were graduate students who were naïve to numerosity comparison experiments.

#### Apparatus

A Macintosh G3 laptop computer was used to generate the display, control the stimuli presentation, and record responses.

#### Approximate comparison task

The purpose of the approximate comparison task was to examine the effect of cumulative element area on accuracy and precision in approximate numerosity comparison among 5–6-year-olds and adults.

#### Design

We applied two experimental conditions: the control and the large element conditions. In the control condition, the size of the cumulative element area and that of the array area in the comparison arrays were identical to those in the standard arrays; in the large element condition, the size of the cumulative element in the standard arrays was larger than that of the comparison arrays. We used the method of constant stimuli in which participants decided in each trial which visual array—the standard array or the comparison array—had more elements. To test the precision of approximate comparison, we obtained Weber fractions. To test the accuracy of the approximate comparison, we derived the PSE. The number of elements in the standard array was 12 and that in the comparison array was either 8, 10, 14, or 16; numerical ratios between the standard and comparison array were 2/3 (pair 8–12), 3/4 (12–16), 5/6 (10/12), 6/7 (12–14), respectively. This range was chosen so that the data points of each participant constructed full psychometric functions.

Each condition had 48 trials (12 repetitions × 4 numerical levels), and resulted in 96 trials in total. Each block had 32 experimental trials and four dummy trials, and there were three blocks in total. Dummy trials were mixed in each block to determine whether the participants understood the task and engaged in the task seriously. The number of dots in the dummy trials in comparison was 2–4, or 4–8, and the 5–6-year-olds could correctly respond if they understood the task. The sequence of the trial was completely randomized in a block. In half the trials, the standard array appeared on the right side, and in the other half it appeared on the left side.

#### Stimuli

**Figure 1:**
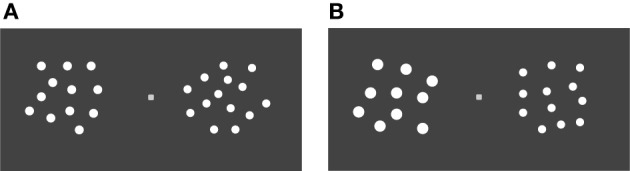
**Schematic view of stimuli in (A) the control condition and (B) the large element condition**.

Schematic views of the stimuli are shown in Figure 1. The stimuli consisted of red or blue dots on a dark gray background. The color of the dots in a particular array was the same, but it differed among arrays. All the dots in a particular array had the same size, but the diameter of the dot in the standard arrays varied among arrays, between 8 arc min to 12 arc min visual angle (9, 10, 11, and 12 visual angle).

In the control condition, in half the trials in a block, the average diameter of dots in the standard array was equal to that in the comparison array area. In the other half of the trials in a block, the diameter of the dot was adjusted such that the summed area of dots in the comparison array was not correlated with the number of stimuli. Thus, the total area of dots was not a reliable indicator of numerosity. In the large element condition, the size of each element of the standard arrays was two times larger than that of the comparison arrays; therefore, the cumulative area of elements in the standard array was approximately two times larger than that in the comparison array. In both conditions, the size of the array area in the comparison and the standard arrays was identical. The array area extended 2.8° × 2.8° visual angle with a viewing distance of approximately 40 cm form the computer monitor. Elements could be located in any given position in a set of positions with *x*-axis and *y*-axis noise. The sets of the position also varied from array to array. We controlled the minimum inter-element distance and the regularity of spatial distribution of elements so that their spatial arrangement was not a reliable cue to numerosity judgments.

#### Procedure

Participants performed all tasks individually in a quiet room. Each participant was seated at a table in front of a laptop computer at a viewing distance of approximately 40 cm. The experimenter was seated on the right side of each participant to ensure that the participants performed the task as instructed. Each trial began with a gray fixation point in the center of the monitor. Two sets of arrays—a standard and a comparison—were displayed on the right or left side of the center of the monitor. Both arrays were displayed simultaneously for 1600 ms, followed by a blank screen. The participant's task was to detect whether the right or the left array contained more dots.

At the beginning of the experimental session, children received a sticker card on which they put their favorite sticker at the end of each block. They were instructed to press one of the six yellow labeled keys on the left if the left array contained more dots, and to press one of the similar six keys on the right if the right array had more dots. At the beginning of each block, participants were instructed to judge by only the number of elements and not by other properties of the elements. The participants were given four practice trials before the experiment. No feedback about correctness of the choices was provided. For 5–6-year olds, pictures that might encourage them to engage in the task were displayed on the screen after every twelve trials in a block.

It took approximately 2–3 min to complete a block. One to two minutes' rest was given between blocks. Adults took about 10 min to finish all blocks. Children, however, took about 15–20 min because they needed time to choose their sticker and put it on their card.

#### Analysis

The data results below the chance level in dummy trials were excluded. The PSE and Weber fractions were measured using the method of constant stimuli. First, the number of dots in comparison arrays was plotted on the *x*-axes, and the proportion of “greater” responses for each comparison array was plotted on the *y*-axes. Then, the plotted data points constructed the psychometric function approximated by a cumulative Gaussian function, on which the difference threshold was obtained. The difference threshold was defined as the smallest amount of the dot number change to achieve 75% correct responses. The Weber fractions were obtained by dividing the difference thresholds by the standard dot numbers. The PSEs were obtained as the value of the location on the psychometric function at which the standard and comparison arrays' choice probabilities were equal to 50%. In this experiment, we obtained the standardized PSE (*PSE*) by dividing the PSE by the number of standard elements.

#### Non symbolic numerical matching task for the assessment of their acquisition of number concept

**Figure 2:**
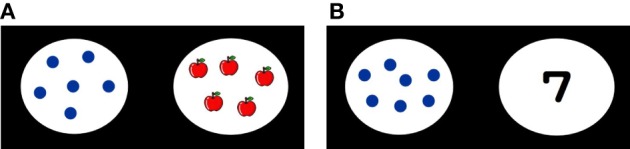
**An example of the stimuli for (A) non-symbolic numerical matching task and (B) symbolic numerical matching task**.

An example of the stimuli of non symbolic numerical matching task is shown in Figure 2A. The numbers of dots and objects pair were either of 4-4, 4-5, 5-5, 5-6, 6-6, 6-7, 7-7, or 7-8. Eight trials were conducted in total. Pictures for the objects differed in each trial. In one half of the trials the numbers of the dots and objects were the same, and in the other half they were different. Each child was asked whether the numbers of the dots and objects were the same or different. If they were the same, children needed to press one of the six yellow labeled keys on the right side. If they were different, they were asked to press red labeled key—the space bar. Children were asked to count the dots and objects one by one out loud or point their finger to the objects and dots. Correct responses for each trial were recorded. The performance of each child was scored from 1 to 4. When children could not respond correctly to any trial, they scored 1; when they made up to four correct responses, they scored 2; when they made up to six correct responses, they scored 3; when they responded correctly to all trials, they scored 4.

#### Symbolic numerical matching task for the assessment of their initial numerical knowledge

In the symbolic numerical matching task, children were asked to answer whether the number of dots and the Arabic numeral were the same or different. An example of the stimuli is shown in Figure 2B. The pairs of the numbers of dots and Arabic numerals were 4-5, 5-5, 6-7, 7-7, 8-9, 9-9, 10-9, and 11-11. There were eight trials in total. In one half of the trials the numbers of the dots and numerals were the same, and in the other half they were different. Each child was asked whether the numbers of the dots and numeral were the same or different. When they were the same, each participant needed to press one of six yellow labeled keys on the right side. When they were not the same, children needed to press the red labeled key—the space bar. Participants were asked to count the dots one by one out loud or point their finger to the dots.

Correct responses and response time for each trial were recorded. The mean correct response ratio and mean response time for each child were calculated. Response times larger or smaller than three standard deviations were excluded. The inverse efficiency score (IE score) was used to assess each child's symbolic numerical knowledge because it combines accuracy and speed, both of which are deemed important in the assessment of numerical ability (e.g., Iuculano et al., 2008). The IE score was calculated for each subject by dividing the adjusted mean response time by the proportion of correct responses.

### Results

#### Approximate comparison task

**Figure 3:**
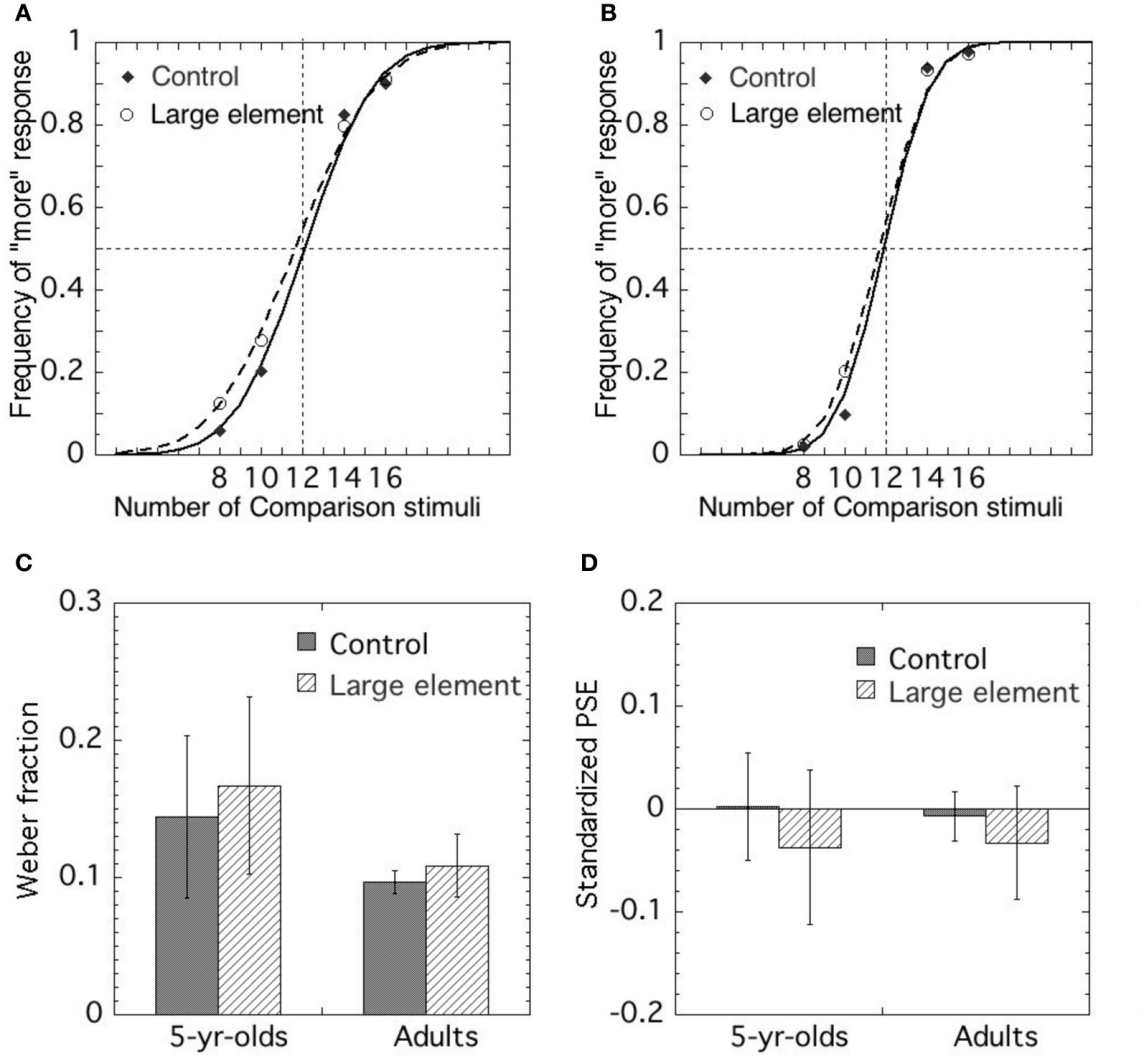
**Choice frequency of more elements as a function of the number of comparison stimuli in (A) 5-year-olds and (B) adults.** Mean Weber fraction **(C)** and standardized PSE **(D)** of each condition in 5-year-olds and in adults. Error bars represent standard deviations.

Figure 3 shows the means of the Weber fractions and *PSEs* in each condition for 5–6-year-olds and adults.

#### 5–6-year-olds

The performance of one participant in the dummy trials was below the chance level, and therefore we excluded that data from further analysis. The fits of data points to psychometric functions were generally good. The Pearson-moment correlation coefficient exceeded 0.9 in all cases, with the exception of one participant in the control condition and two in the large element condition. The data for these participants were excluded, but those of the remaining participants were used for further analysis.

The precision of the approximate comparison was assessed by Weber fractions. The means of Weber fractions in the control and large element conditions were 0.146 (*SD* 0.06) and 0.174 (*SD* 0.09), respectively. We conducted a *t*-test to compare the mean of the Weber fractions of each condition. The results demonstrated that the mean of the Weber fractions in the large element condition was significantly larger than that in the control condition [*t*_(16)_ = −2.486, *p* < 0.05, *r* = 0.53], suggesting that the precision in the large element condition was lower than that in the control condition.

To assess the accuracy in approximate comparison, we examined the *PSEs* in each condition and compared them. The means of *PSEs* in the control and large element conditions were 0.002 (*SD* 0.05) and −0.037 (*SD* 0.07), respectively. To test whether a systematic deviation occurred from the objective value (i.e., *PSE* value of 0), we conducted a one-sample *t*-test to compare the mean of the *PSEs* in each condition to the *PSE* of 0. In the large element condition, significant difference was found between the mean of the *PSEs* and 0 [*t*_(16)_ = −2.437, *p* < 0.05, *r* = 0.52], suggesting that the number of larger elements was underestimated, whereas no significant difference was observed in the control condition [*t*_(17)_ = −0.233, *p* > 0.1].

#### Adult

The fits of data indicate that psychometric functions were generally good, and the Pearson moment correlation coefficient exceeded 0.9 in all cases. Precision of approximate comparison was assessed by Weber fractions. The means of Weber fractions in the control and large element conditions were 0.10 (*SD* 0.01) and 0.11 (*SD* 0.02), respectively. We conducted a *t*-test to compare the means of the Weber fractions of each condition and found that the mean in the large element condition was significantly larger than that in the control condition [*t*_(15)_ = −2.424, *p* < 0.05, *r* = 0.53], suggesting that the precision in the large element was lower than that in the control condition.

To assess the accuracy in approximate comparison, we examined the *PSEs* in each condition and compared them. The means of *PSEs* in the control and the large element conditions were −0.006 (*SD* 0.02) and −0.024 (*SD* 0.06), respectively. To test whether a systematic deviation occurred from the objective value (i.e., PSE value of 0), we conducted a one-sample *t*-test to compare the mean of the *PSEs* in each condition to the *PSE* of 0. In the large element condition, significant difference were found between the mean of the *PSEs* and the *PSE* of 0 [*t*_(15)_ = −2.401, *p* < 0.05, *r* = 0.53], suggesting that the number of larger elements was underestimated. However, no significant difference was observed between those in the control condition [*t*_(15)_ = −1.157, *p* > 0.1].

#### Comparison of 5–6-year-olds and adults

We compared the precision in approximate comparison for the 5–6-year-olds and the adults in each condition. The Welch *t* test for independent samples indicated that the mean Weber fraction for the adults was significantly lower than that for the 5–6-year-olds in the control condition [*t*_(18)_ = −3.955, *p* < 0.01, *r* = 0.68] and the large element condition [*t*_(19)_ = −4.154, *p* < 0.01, *r* = 0.69]. Thus, the results suggest that the performance of adults was more precise than that of 5–6-year-olds.

We compared the size of bias for 5–6-year-olds and adults in each condition. The Welch *t* test for independent samples indicated that the mean *PSEs* for the 5–6-year-olds was not significantly different from those of the adults in the control condition [*t*_(25)_ = −0.564, *p* > 0.05] and the large element condition [*t*_(30)_ = 0.314, *p* > 0.05]. Thus, the results suggest that there was no difference in the size of bias between the 5–6-year-olds and the adults.

#### Acquisition of the numerical concept

In the non symbolic numerical matching task, three out of 20 children scored 3, and rest of children scored 4. Therefore, it could be assumed that all the children participating in the present study had acquired numerical concepts and exact counting.

#### Relationship between approximate numerosity and the symbolic numerical knowledge

**Figure 4:**
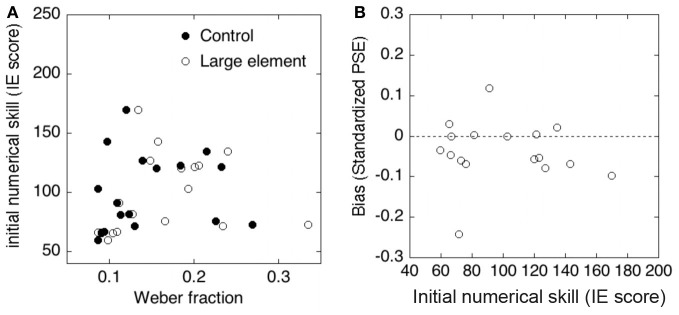
**(A)** Correlations between the Weber fraction and initial numerical skill (inverse efficiency scores) in the control and large element conditions. **(B)** Correlation between the initial numerical skill and the standardized PSE (i.e., size of bias).

To examine whether a relationship existed between approximate comparison and the symbolic numerical knowledge, we performed correlation analysis between the results of the approximate comparison task and the symbolic numerical matching task. Figures 4A,B show the results of each analysis. First, we tested the correlation between the precision of approximate numerosity and the initial numerical skill. No significant correlation between the Weber fraction and IE scores was observed in either the control condition (*r* = 0.17, *p* > 0.1) or the large element condition (*r* = 0.17, *p* > 0.1). Second, we examined the relationship between the accuracy of approximate numerosity and the initial numerical skill. No significant correlation between *PSEs* in approximate comparison and IE score was observed (*r* = −0.07, *p* > 0.1).

### Discussion

The results demonstrate three significant findings concerning the effect of the cumulative element area on the approximate numerosity comparison. First, as shown in the larger Weber fraction in the large element condition, precision deteriorated when the size of the element was manipulated both in 5–6-year-olds and adults. Second, as shown in the PSE, the number of elements in the large element condition revealed the bias that caused the larger elements to be judged less numerous than the smaller ones. Third, the precision of the approximate numerosity comparison in adults was higher than that in 5–6-year-olds, replicating the results of previous research (Halberda and Feigenson, 2008).

Overall, the results in study 1 have clearly demonstrated that the large element affects the precision and accuracy in approximate comparison both in 5–6-year-olds and adults. The results were consistent with the previous research in adults (Shuman and Spelke, 2006; Tokita and Ishiguchi, 2010).

We also tested whether approximate comparison correlated with initial numerical skill in 5–6-year-olds. The results show that precision and the size of bias in approximate comparison does not correlate with initial numerical skill. The result was consistent with Soltesz et al.'s (2010) previous research that demonstrated that counting knowledge did not correlate with approximate numerosity comparison.

## Study 2

We examined whether and how the manipulation of the array area affects the numerosity comparison. Following the comparison task, 5–6-year-olds performed the non symbolic numerical matching task and the symbolic numerical matching task in the same way as those in Study 1.

### Method

#### Participants

Twenty children (mean age 5.8 years, *SD* = 0.30 years; range 5.5–6.3 years; 11 females, 9 males) and sixteen adults (mean age 27.7 years, *SD* = 4.35 years; range 23–40 years; 12 females, 4 males) participated. Informed consents from a parent of each child were obtained prior to the experiment. None had a statement of special educational needs. All children were recruited though kindergartens and received a sticker to thank them for taking part.

The stimuli, apparatus, procedure, and analysis were the same as those in Study 1, with the following exceptions.

#### Design

We applied two experimental conditions: the control condition and the large array area conditions.

#### Stimuli

**Figure 5:**
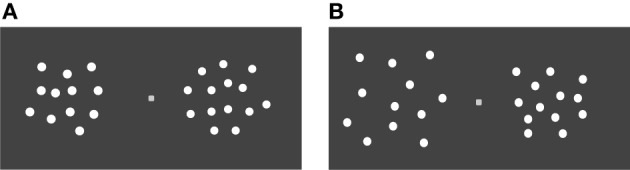
**Schematic view of stimuli in (A) the control and (B) the large array area condition**.

Schematic views of the stimuli are shown in Figure 5. The stimuli in the control condition were the same as those in Study 1. In the large array area condition, the size of the array area of the standard arrays was two times larger than that of the comparison arrays; therefore, the display area of the standard arrays was 4° × 4° visual angle with a viewing distance of 40 cm form the computer monitor.

Following the approximate comparison task, 5-year-olds performed the non symbolic numerical matching task and the symbolic numerical matching task the same as those in Study 1.

### Results

#### Approximate comparison task

**Figure 6:**
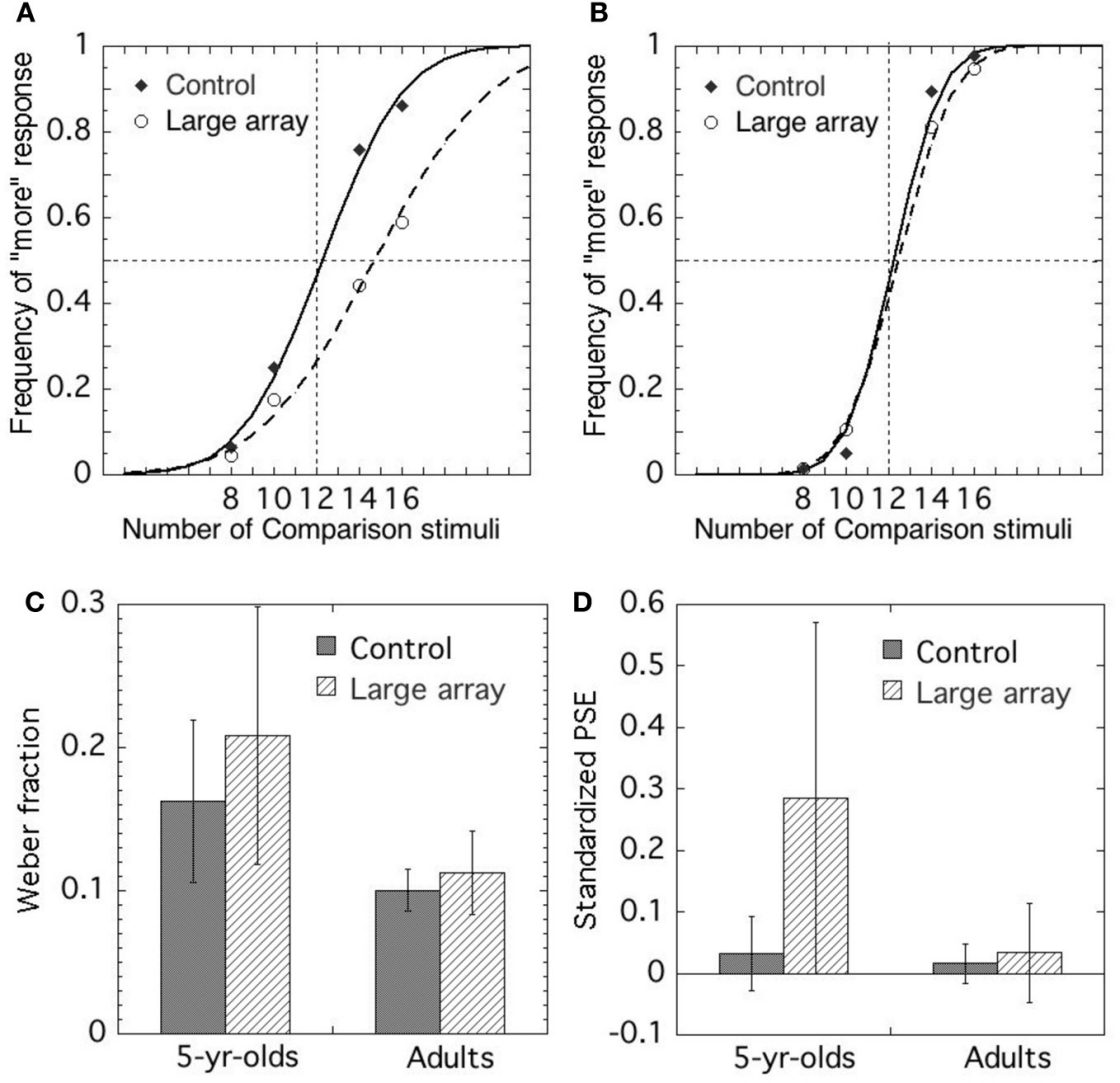
**Choice frequency of more elements as a function of the number of comparison stimuli in (A) 5-year-olds and (B) adults.** Mean Weber fraction **(C)** and standardized PSE **(D)** of each condition in 5-year-olds and in adults. Error bars represent standard deviations.

Figure 6 shows the means of the Weber fractions and the means of *PSE*s in each condition for 5–6-year-olds and adults.

#### 5–6-year-olds

The performance of one participant in the dummy trials was below the chance level, and therefore, we excluded the data for further analysis. The fits of data points to psychometric functions were generally good, and the Pearson-moment correlation coefficient exceeded 0.9 in all cases with the exception of one participant in the control condition, and three in the large array area condition. The data for these participants were excluded, but those of the remaining participants were used for further analysis.

The precision of the approximate comparison was assessed by Weber fractions. The means of Weber fractions in the control and the large array area conditions were 0.163 (*SD* 0.06) and 0.208 (*SD* 0.09), respectively. We conducted a *t*-test to compare the mean of the Weber fractions of each condition. The results demonstrated that the mean of the Weber fractions in the large array area condition was significantly larger than that in the control condition [*t*_(15)_ = −2.310, *p* < 0.05, *r* = 0.51], suggesting that the precision in the large array area condition was lower than that in the control condition.

To assess the accuracy in approximate comparison, we examined the *PSEs* in each condition and compared them. The means of *PSEs* in the control and the large array area conditions were 0.04 (*SD* 0.06) and 0.27 (*SD* 0.29), respectively. To test whether there was a systematic deviation occurred from the objective value (i.e., *PSE* value of 0), we conducted a one-sample *t*-test to compare the mean of *PSEs* in each condition to the *PSE* of 0. In the large array area condition, significant difference was found between the mean of *PSEs* and *PSE* of 0 [*t*_(16)_ = −2.437, *p* < 0.05, *r* = 0.52], suggesting that the number of elements in larger array area was overestimated, whereas no significant difference was observed between those in the control condition [*t*_(17)_ = −0.233, *p* > 0.1].

#### Adults

The fits of data points to psychometric functions were generally good, and the Pearson-moment correlation coefficient exceeded 0.9 in all cases. The precision of the approximate comparison was assessed by Weber fractions. The means of Weber fractions in the control and the large array area conditions were 0.10(*SD* 0.015) and 0.11 (*SD* 0.029), respectively. We conducted a *t*-test to compare the mean of the Weber fractions of each condition and found that the mean of Weber fractions in the large array area condition was significantly larger than that in the control condition [*t*_(15)_ = −2.846, *p* < 0.05, *r* = 0.59], suggesting that precision in the large array area condition was lower than that in the control condition. To assess the accuracy in approximate comparison, we examined the *PSEs* in each condition and compared them. The means of *PSEs* in the control and the large array area conditions were 0.026 (*SD* 0.032) and 0.033 (*SD* 0.081), respectively. To test whether a systematic deviation occurred from the objective value (i.e., *PSE* value of 0), we conducted a one-sample t-test to compare the mean of *PSEs* in each condition to *PSE* of 0. No significant difference was found between the mean of the *PSEs* and 0 in the control condition [*t*_(15)_ = 2.042, *p* > 0.05, *r* = 0.47] and in the large array area condition [*t*_(15)_ = 1.658, *p* > 0.05, *r* = 0.39].

In addition, we compared the absolute values of *PSEs* in the control and the large array area conditions to demonstrate that the absolute values of *PSEs* in the large array area condition was significantly larger than those in the control condition [*t*_(15)_ = −2.143, *p* < 0.05, *r* = 0.49].

#### Comparison of 5–6-year-olds and adults

We compared the precision in numerosity comparison for 5–6-year-olds and adults in each condition. The Welch *t* test for independent samples indicated that the mean Weber fraction for 5–6-year-olds was significantly lower than that for adults in the control condition [*t*_(16)_ = −5.266, *p* < 0.01, *r* = 0.80] and the large array area condition [*t*_(17)_ = −4.455, *p* < 0.01, *r* = 0.76]. Thus, the results suggest that the performance of adults was more precise than that of 5–6-year-olds in approximate numerosity comparison. We compared the size of bias for 5–6-year-olds and adults in each condition. The Welch *t* test for independent samples indicated that the mean standardized *PSE* for the 5–6-year-olds was not significantly different from those of the adults in the control condition [*t*_(16)_ = −0.966, *p* > 0.05], whereas the mean of *PSEs* was significantly greater for the 5–6-year-olds than for the adults in the large array area condition [*t*_(17)_ = −3.377, *p* < 0.01, *r* = 0.63]. Namely, the 5–6-year-olds demonstrated the greater bias than adults in the large array area condition.

#### Acquisition of numerical concept

In the non symbolic numerical matching task, 2 out of 20 children were scored 3 and rest of children scored 4. Therefore, it could be assumed that all the children participating in the present study had acquired numerical concepts and exact counting skill.

#### Relationship between approximate numerosity and the symbolic numerical knowledge

**Figure 7:**
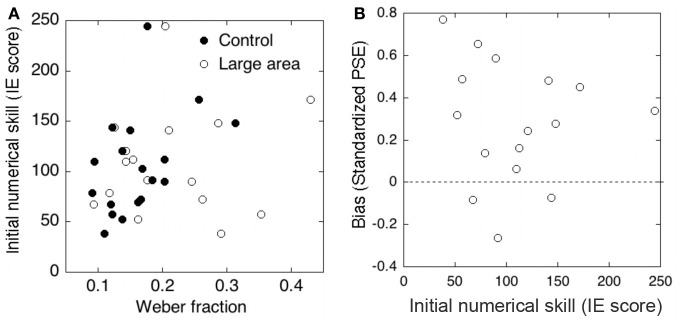
**(A)** Correlations between the Weber fraction and the initial numerical skill (inverse efficiency scores) in the control and large element conditions. **(B)** Correlation between the initial numerical skill and the standardized PSE (i.e., size of bias).

To examine whether a relationship existed between the performance of approximate comparison and the initial numerical skill, we performed correlation analysis between the results of approximate comparison task and the symbolic numerical matching task. Figures 7A,B show the results of each analysis. First, we tested the relation between the precision of approximate numerosity and the initial numerical skill. No significant correlation between the Weber fraction and IE scores was observed in the control condition (*r* = 0.44, *p* > 0.1) or in the large array area condition (*r* = 0.10, *p* > 0.1). Second, we examined the relationship between the accuracy of approximate numerosity and the initial numerical skill. No significant correlation between standardized *PSEs* in approximate comparison and IE score was observed (*r* = −0.11, *p* > 0.1).

### Discussion

The results demonstrate three significant findings concerning the effect of the array area on the approximate numerosity comparison. First, as shown in the larger Weber fraction in the large array area condition, precision deteriorated when the array area was manipulated both in 5–6-year-olds and adults. Second, as shown in *PSEs*, the number of elements in the large array area condition revealed the bias that caused the number of elements in the large array area judged more numerous than those in the control condition for 5–6-year-olds. No systematic bias was observed in adults, however, the absolute value of *PSE*s was significantly larger in the large array area condition than those in the control condition. Third, precision of the approximate numerosity comparison in adults was higher than that in 5–6-year-olds, replicating the result of study 1 and of previous research (Halberda and Feigenson, 2008). Over all, the results in Study 2 have clearly demonstrated that the array area affects the precision and accuracy in approximate comparison both in 5–6-year-olds and adults. We also tested whether the approximate comparison correlated with the initial numerical skill in 5–6-year-olds. The results show that precision and the size of bias in approximate comparison does not correlate with initial numerical skill as the same as those in Study 1.

## General discussion

Despite a larger number of studies, it remains unclear whether and how perceptual variables affect approximate numerical judgment across development. Certain studies have claimed that even infants can make approximate numerical judgments irrespective of the perceptual variables such as element area, contour length, luminance, and spatial arrangements of elements. Others have suggested that infants and young children are easily affected by the perceptual variables in numerical judgments because they incorporate the information other than the number of elements when they try to abstract numerical values. In an attempt to reconcile this discrepancy, we examined whether and how perceptual variables (i.e., size of the element area and array area) affect the approximate numerosity comparison in 5–6-year-olds and adults using the identical experimental procedure. In the assessment of the effect of the perceptual variables, we measured precision and accuracy of the numerosity comparison by applying the psychophysical method. Overall, our results show that the perceptual variables affected the numerosity comparison such that precision and accuracy deteriorated when the perceptual variables were manipulated in both 5–6-year-olds and adults.

In Study 1, we tested how the manipulation of the cumulative element area would affect approximate numerosity comparison. The results showed that Weber fractions increased in the large element condition, reflecting lower precision. For accuracy, the larger elements were judged less numerous than the smaller elements. The only difference between 5–6-year-olds and adults was that the mean Weber fraction for 5–6-year-olds was larger than that for adults, suggesting that precision in approximate comparison was significantly higher in adults.

In study 2, we tested how the manipulation of the array area would affect the precision and accuracy of approximate numerosity comparison. The results demonstrated that Weber fractions increased in the large array condition, reflecting lower precision. For accuracy, the number of elements in the large array area was judged more numerous by 5–6-year-olds. Two differences were found between 5–6-year-olds and adults. First, precision in approximate comparison was significantly higher in adults than in 5–6-year-olds, the same as the result of study 1. Second, size of bias observed in the array area condition was much larger for 5–6-year-olds than for adults. Interestingly, this result is consistent with Piaget's finding that children tend to judge the number of objects in a larger spread area as more numerous than those in a smaller spread area.

The findings that perceptual variables affect approximate comparisons are largely consistent with those of previous research involving adult participants with no extensive practice (e.g., Krueger, 1984; Ginsburg and Nicholls, 1988; Shuman and Spelke, 2006; Sophian and Chu, 2008; Tokita and Ishiguchi, 2010; Gebuis and Reynvoet, 2012). Thus, on the basis of previous research and the present study, we can conclude that the effect of perceptual variables on 5-year-olds is qualitatively similar to that on adults. The fact that both children and adults demonstrated a higher variability of precision and accuracy in the condition with manipulated perceptual variables suggests that they did not make numerosity judgments solely on the basis of the numerical information but by incorporating perceptual factors such as the size of cumulative element area, size of array area, and spatial arrangements. That is, the ability to represent approximate numerosity may not be determinant but easily altered by manipulation of the perceptual features of elements in a set or the environment in which the elements are laid out. Further research is needed to determine the processing level at which the perceptual variables affect the judgment of numerosity. It also needs to be studied how they cause the deterioration of the precision and accuracy and how that effect would change during development through the school years.

In addition, we examined the relationship between approximate numerosity comparison and symbolic numerical knowledge in 5–6-year-olds. We tested two types of correlations: the correlation between the precision of approximate comparison and symbolic numerical knowledge and that between the accuracy of approximate comparison and symbolic numerical knowledge. The results demonstrate that both precision and accuracy in approximate comparison did not relate to the symbolic numerical knowledge in Study 1 as well as in Study 2. Several studies have found that young children with little or no knowledge of numerical concepts and exact counting tend to be affected by perceptual variables such as a large presentation area. However, our result suggests that the effect of perceptual variables was not related to symbolic numerical knowledge. Conversely, even children with exact counting knowledge tend to overestimate or underestimate numerical values in given perceptual conditions, as do adults. These results support the finding of Iuculano et al. (2008) and Soltesz et al. (2010). The former demonstrated that approximate number tasks such as approximate comparison and arithmetic were not related to the symbolic number comparison and exact addition in 8- and 9-year-old children, implying that the system for approximate numerosities has little or nothing to do with the system for exact numerosities. It should be noted that the recent study by Lourenco et al. (2012) claims the relation between mathematical ability and sensitivity in numerical estimation, demonstrating that individual differences in both number and cumulative area precision in their magnitude comparison task correlated with inter-individual variability in math competence. One possible reason for the different result between the present study and Lourenco's is how numerical knowledge has been tested: the present study assessed the initial number knowledge such as counting, while the Lourenco assessed advanced mathematical skills including arithmetic and geometry. Extensive research is necessary to reveal the relation between numerical sensitivity and numerical knowledge across development stages.

Our results may challenge the claim that humans are equipped with a dedicated mechanism for approximate numerosity representation. The findings, together with previous research on the effect of perceptual variables on approximate numerosity comparison, suggest that people tend to use multiple perceptual cues in the process of extracting numerical information. The perceptual cues could be element area, array area, spatial arrangement of elements in a set, luminance, etc. Why is it necessary to integrate multiple perceptual cues if we are equipped with a dedicated system for approximate numerosity? In the argument for the approximate numerosity system, there might be confusion between the process's final result (approximate numerical representation) and the process used to extract numerical information from the visual array. The number representation may be abstract, but the process of abstracting the numerical values may not be. In the processes of abstracting numerical values from the visual array, one must use perceptual information to define elements, integrate the information, and derive a number representation for the visual array. Conversely, the process may not yield accurate responses because of attentional, memory, or resolutional limitation. The fact that the precision in numerosity comparison was higher in adults than in 5-year-olds could be explained by the difference in their cognitive abilities such as attention and working memory improve; thus, the precision and accuracy in numerosity comparison may improve across development.

In conclusion, we provide evidence for the effects of perceptual variables upon numerosity processing in both 5–6-year-olds and adults. We also demonstrate that least relationship exists between approximate comparison and initial numerical skill in 5–6-year-olds. These findings may challenge the claims of a dedicated system for approximate numerosity representation. To further explore the nature of numerical representation, we need to examine how people extract numerical information in the image or sequence of sounds presented though sensory modalities, how people use their perceptual, attentional, and memory abilities in extracting numerical information, and how the processes may be expanded across development.

### Conflict of interest statement

The authors declare that the research was conducted in the absence of any commercial or financial relationships that could be construed as a potential conflict of interest.
